# Growth mindset and intrapersonal dimensions of social emotional skills: mediating roles of negative automatic thoughts and self-control in elementary students

**DOI:** 10.3389/fpsyg.2026.1714395

**Published:** 2026-03-13

**Authors:** Ganzhen Ding, Wenbo Wang, Weidong Tao

**Affiliations:** 1 Department of Psychology, School of Education, Huzhou University, Huzhou, China; 2 Nanjing University of Vocational and Technical Education, Nanjing, China

**Keywords:** elementary school students, growth mindset, indifferent mindset, negative automatic thoughts, self-control, social emotional skills

## Abstract

**Introduction:**

Although previous research has linked growth mindset to the intrapersonal dimensions of social emotional skills, little is known about how different mindset types relate to these skills. Moreover, the role of negative automatic thoughts in this relationship among Grade 5 and Grade 6 elementary students remains unclear.

**Methods:**

This study examined the direct and indirect associations between growth mindset and the intrapersonal dimensions of social emotional skills among fifth- and sixth-grade elementary students. A total of 541 students participated in the study.

**Results:**

The analysis showed that growth mindset was directly associated with the intrapersonal dimensions of social emotional skills and indirectly linked through negative automatic thoughts and self-control. These associations were stronger in the growth mindset group compared with the indifferent mindset group, indicating different patterns across mindset groups.

**Discussion:**

These findings shed light on the mechanisms that link growth mindset with intrapersonal social emotional skills in elementary students.

## 1 Introduction

Social emotional skills play a critical role in an individual’s ability to recognize and regulate emotions, understand own and others’ emotional states, establish and maintain positive relationships, and make responsible decisions in social situations (Elias and Arnold, 2006; Elias et al., 1997; Taylor and Larson, 1999). It includes five core skills: self-awareness, self-management, social awareness, relationship skills, and responsible decision-making (CASEL, 2023; Lemerise and Arsenio, 2000). Social emotional skills have been associated with reduced problematic behaviors (Yang et al., 2020). More importantly, evidence has shown that social emotional skills are a key part of learning and academic success (Domitrovich et al., 2017; McKown et al., 2015). Given its critical role in educational settings, this study examines social emotional skills as the primary outcome variable to investigate their underlying cognitive and belief- based mechanisms. Social emotional skills development during childhood has been linked to multiple life outcomes (Davidson and Begley, 2012; Liew, 2012) and is considered important for wellbeing, educational attainment, emotional stability, and social integration (Jones et al., 2015).

Although previous research has established a link between growth mindset and social emotional skills (Huang et al., 2023; Jiang et al., 2024; Zhang et al., 2025a; Zhang et al., 2025b), it has predominantly treated mindset as a continuous variable, overlooking the potential distinctiveness of mindset types (e.g., indifferent mindset). Furthermore, despite the acknowledged importance of social emotional skills, the specific pathways through which mindset types influence these skills—via cognitive regulators like negative automatic thoughts—remain underexplored among upper elementary students.

### 1.1 Social emotional skills and interventional studies

Social emotional skills are consistently linked to enhanced academic performance, including an average 11-percentile-point gain in achievement (Durlak et al., 2011), as well as improved mental health and prosocial behaviors (Greenberg et al., 2003; Huang, 2015). Subsequent research, including recent large-scale meta-analyses, confirms that these academic and developmental benefits are robust and can be sustained years after intervention (Cipriano et al., 2023; Taylor et al., 2017). Moreover, social emotional skills programs have been associated with prosocial behaviors and a sense of satisfaction and belonging in community involvement (Greenberg et al., 2003; Hawkins et al., 2004).

### 1.2 Growth mindset and its associations

Growth mindset is characterized by an individual’s belief in the malleability of core attributes such as intelligence, abilities, and personality (Dweck, 2006). In contrast, a fixed mindset is the belief that abilities or personality are innate and unchangeable. Individuals with a growth mindset typically establish learning-oriented goals, embrace challenges, and demonstrate greater resilience and perseverance when facing setbacks (Dweck and Leggett, 1988; Yeager and Dweck, 2012).

Research indicates that growth mindset interventions have been associated with improvements in academic achievement (Blackwell et al., 2007; Yeager et al., 2019), mental health (Burnette et al., 2020b; Schleider and Weisz, 2018; Zhu et al., 2023), wellbeing (Ortiz Alvarado et al., 2019; Zhao et al., 2021), and social emotional learning (Burnette et al., 2020a). This approach has been reported to be associated with improved performance among college students (Canning et al., 2019, 2022) and reduced stereotype threat in some studies (Aronson et al., 2002; Spitzer and Aronson, 2015).

Although the benefits of growth mindset are well-documented, some studies have presented inconsistent findings. For instance, Li and Bates (2019) found no association between growth mindset and academic achievement (measured by GPA in core subjects) in a sample of 433 Chinese primary school students, based on data from two studies conducted in grades 5–6. According to the 2018 PISA assessment (Sun et al., 2021), mainland Chinese students are more likely than their U. S. counterparts to hold fixed mindsets about intelligence, and unlike in Western contexts where a positive link exists, the association between growth mindset and academic performance in China is unreliable or even slightly negative, suggesting that these effects are highly context- dependent. Yeager and Dweck (2020) affirmed a replicable link between growth mindset and academic achievement, particularly in adversity and failure. However, they acknowledged heterogeneity in results, noting cultural impacts on academic performance and social emotional skills, especially in collectivist cultures such as China (Costa and Faria, 2018).

Current research has begun to move beyond a simple binary view of mindset (high growth vs. fixed). Chiu et al. (2023) introduced the concept of an ambivalent mindset alongside the traditional fixed and growth mindsets. Building on this person-centered approach, it is reasonable to identify individuals who hold neither mindset strongly—an “indifferent mindset” (low growth and low fixed). In contrast to growth or fixed mindsets, individuals with an indifferent mindset may lack a coherent belief system regarding their attributes, potentially leading to passivity or disengagement. However, the existing literature rarely examines this “indifferent” subgroup, particularly how an absence of distinct mindset beliefs interacts with negative cognitions and social emotional competencies.

The support for growth mindset interventions in elementary school students is not as robust (Dweck, 2012). Most existing studies focus primarily on academic outcomes, such as reading and writing skills, rather than examining broader developmental domains. Notably, the initial meta-analysis on growth mindset interventions in elementary students included only 10 papers (Savvides and Bond, 2021), and few of these addressed outcomes beyond academics. Thus, there remains a significant lack of research exploring the intersection between growth mindset and social emotional skills in this age group.

### 1.3 Implicit theories of emotion

To explain how growth mindset connects to social emotional skills and to better understand the differences between mindset groups, we use the idea of implicit theories of emotion. Although Dweck’s theory shows that beliefs about intelligence and personality are malleable and can be developed through experience (Dweck, 2006), the relevance of this mindset to social emotional skills depends on the extent to which this belief system extends to the emotional domain. Tamir et al. (2007) introduced the concept of implicit theories of emotion, noting that individuals who endorse an emotional entity theory—believing that emotions are fixed and unchangeable—tend to report more negative emotions, reduced social support, and lower emotional wellbeing. Burnette et al. (2013) observed a connection between growth mindset and enhanced self- regulation and self-control abilities. These self-control abilities have been associated with subsequent changes in GPA (Duckworth and Gross, 2014; Duckworth et al., 2019).

Social emotional learning interventions typically employ either skill-based approaches to teach specific abilities, or atmosphere-based approaches to foster supportive environments (Osher and Berg, 2017). Yeager (2017) argues that effective social emotional skill intervention programs should tap into adolescents’ developmental motivations and shape their attitudes and beliefs. Examining growth mindset in upper elementary students helps elucidate the internal cognitive factors driving social emotional development prior to adolescence. This suggests that acquiring social emotional skills is not solely dependent on external factors like teacher-led or environmental support. Rather, it is heavily influenced by students’ own adaptive belief systems (Rissanen et al., 2021).

### 1.4 Negative automatic thoughts and its mediating role

Negative automatic thoughts (Beck, 1976, 2021; Iancu et al., 2015; Watkins, 2008) are immediate, uncontrollable negative evaluations of oneself, the environment and future. These thoughts can manifest in various forms, such as overgeneralization, magnification/minimization, dichotomous thinking, self-blame, catastrophizing, and discounting positive experiences (Beck, 1976; Clark and Beck, 2010). Previous studies have broadly linked negative automatic thoughts to psychological distress and maladaptive behaviors in adolescents, including anxiety, depression, and social difficulties (e.g., Choon et al., 2015; Flouri and Panourgia, 2014; Yu et al., 2023). These negative experiences may be negatively associated with the development of students’ social emotional skills (Carter et al., 2006; Chapman et al., 2004; McMahon et al., 2003; Tiet et al., 1998). Research on negative automatic thoughts has primarily focused on college students and patients with psychiatric problems (Crandell and Chambless, 1986; Hollon and Kendall, 1980), with limited focus on pre-adolescence elementary school students.

Cognition plays a significant role in anxiety interventions, as negative cognitive errors and biases contribute to various emotional and behavioral problems (Beck, 1976; Leung and Poon, 2001). Negative automatic thoughts are not only positively associated with social anxiety (Calvete et al., 2013) but also linked to achievement motivation, goal orientation, and attention levels (Knouse et al., 2023). Conceptually, this aligns with a fixed mindset; both involve maladaptive self-evaluations, negative emotions, and cognitions reflecting self-doubt and worthlessness (e.g., “I cannot do it”).

Cognitive-behavioral therapy suggests that automatic thoughts are profoundly shaped by deeper core beliefs or “meaning systems” (Beck, 1976; Lin, 2023). Compared to a fixed mindset, a growth mindset is thought to be part of ‘meaning systems’ that are linked to these automatic thoughts. Therefore, social emotional skills may be associated with automatic thoughts, which are in turn linked to an individual’s growth mindset. However, few studies have yet revealed the intrinsic relationship and specific statistical pathways between these two concepts.

### 1.5 Self-control and its mediating role

Self-control is closely related to cognitive, emotional, and behavioral aspects of functioning. Timpano and Schmidt (2013) define self-control as the conscious ability to adjust behavior based on personal values and societal norms. Research consistently shows a strong connection between self-control and improved psychological wellbeing, including lower levels of depression, anxiety, and stress, as well as an enhanced overall sense of wellness (Tangney et al., 2004). Moreover, higher self-control has been associated with lower engagement in behaviors such as smoking, drinking, and overeating (Duckworth and Seligman, 2005; Hofmann et al., 2012). Self-control is associated with emotion regulation capacities and with lower negative emotional responses (Gross and John, 2003). Therefore, self-control is widely recognized as fundamental in personal growth and development.

Self-control also plays a pivotal role in individual decision-making and behavior (Knoch and Nash, 2015). It is associated with a broad range of outcomes in physical and mental wellbeing, academic and professional contexts, interpersonal relationships, and societal integration (Moffitt et al., 2011). Self-control dynamically influences behavior by helping individuals prioritize long-term goals over implicit, impulsive reactions (Tang et al., 2015).

While certain environments may trigger self-control, achieving greater rewards often requires increased effort and sacrifices. Individuals experiencing negative automatic thoughts, such as feelings of inadequacy and helplessness, may tend to give up, seeing effort and sacrifice as insurmountable obstacles. Cognitive-behavioral interventions, such as exposure therapy, interval schedules, impulse control training, and mindfulness training, are effective in enhancing self-control. Additionally, adopting a growth mindset has been proposed as a cognitive approach that may be associated with stronger self-control (Yuan et al., 2024). Research indicates that growth mindset is associated with self-regulation (Burnette et al., 2013). Scholars propose a practical framework that considers self-regulation, self-efficacy, and self- respect as subcomponents of a growth mindset (Ku and Stager, 2022). Individuals with a growth mindset show increased perseverance in challenging tasks, potentially linked to higher levels of self-control (Chen et al., 2020).

### 1.6 The present study

This study aimed to explore the connections between growth mindset, negative automatic thoughts, self-control, and intrapersonal dimensions of social emotional skills in fifth and sixth-grade students. Specifically, to fill the gaps, this study posed three primary research questions:

1. Is growth mindset associated with the intrapersonal dimensions of social emotional skills among older elementary students?
2. Do negative automatic thoughts and self-control help explain the association between growth mindset and the intrapersonal dimensions of social emotional skills?
3. Do distinct mindset types (such as the indifferent mindset) exhibit different patterns of social emotional skills? The hypotheses were as follows: (1) growth mindset would be associated with fewer negative automatic thoughts and with higher self-control; (2) negative automatic thoughts would statistically mediate the association between growth mindset and the intrapersonal dimensions of social emotional skills in the cross- sectional model; (3) self-control would statistically mediate the association between growth mindset and the intrapersonal dimensions of social emotional skills in the cross- sectional model; (4) negative automatic thoughts would be negatively associated with self-control; (5) negative automatic thoughts and self-control would sequentially mediate the statistical association between growth mindset and social emotional skills in the cross-sectional model. And (6) growth mindset group will show higher levels of self-control and the intrapersonal dimensions of social emotional skills than the indifferent mindset group.

## 2 Methods

### 2.1 Participants

Participants were recruited from four public elementary schools in the urban district of Huzhou, China, as part of a school-based mental health education program. Using convenience sampling, data were collected in March 2022, with 564 fifth- and sixth-grade students initially completing paper-and-pencil questionnaires. After excluding invalid responses (e.g., consistently selecting the same option or submitting a blank questionnaire), a total of 541 valid questionnaires were retained, yielding a response validity rate of 95.9%.

The final sample consisted of 262 male (48.4%) and 279 female students (51.6%). Of these participants, 334 were in the fifth grade and 207 were in the sixth grade, with an average age of 10.8 years (range: 9–13 years). Given the potential influence of recent shifts in China’s family planning policies (i.e., the two-child and three-child policies), family size was recorded: students from single-child families accounted for 35.3% of the sample, while those with one sibling accounted for 58.4%. Additionally, students self-reported their academic abilities as weak (23.9%), average (45.8%), or strong (30.3%), and their family’s socioeconomic status as low (41.9%), medium (49.0%), or high (9.1%).

### 2.2 Measures

#### 2.2.1 Growth mindset

Growth mindset was assessed using the Implicit Theories of Intelligence Scale (Dweck, 1999), a valid, authoritative tool measuring students’ growth mindset. The scale was revised into Chinese by Zhao et al. (2022). It consists of six items divided into two dimensions: fixed mindset and growth mindset. Three items are associated with fixed mindset (e.g., “Intelligence is difficult to change”), while three with growth mindset (e.g., “You can always substantially change your level of intelligence”). Participants rate their agreement with these statements on a six-point Likert scale (1 = strongly disagree, 6 = strongly agree). The fixed mindset items are reverse scored. The mean scores for all six items are calculated, with higher scores indicating a higher level of growth mindset. Following prior studies using midpoint-based classification for mindset categorization (Xu et al., 2022; Zhang et al., 2020), a theoretical midpoint of 3.5 on the six-point Likert scale was adopted as the cutoff value. Specifically, a final average score of <3.5 was considered indicative of a fixed mindset, whereas a score of 3.5 or above reflected a growth mindset. The Cronbach’s alpha for the fixed mindset dimension was 0.73, and for the growth mindset dimension, 0.86, indicating good internal consistency.

#### 2.2.2 The automatic thoughts scale

The Automatic Thoughts Scale developed by Schniering and Rapee (2002) and revised into Chinese by Sun et al. (2015) was utilized to evaluate children’s automatic thoughts related to personal failure (e.g., “I am a failure”) and physical threat (e.g., “I’m going to get hurt”). This assessment exhibits excellent psychometric characteristics, with each subscale differentiating between diverse types of child psychopathology (Schniering and Lyneham, 2007). The scale consists of 20 items scored from 0 (none of the time) to 4 (most of the time). Higher scores indicate a greater frequency of automatic thoughts. The personal failure subscale demonstrated a Cronbach’s alpha of 0.93, while the interpersonal threat subscale, 0.91.

#### 2.2.3 The self-control scale

The Self-Control Scale was initially developed by Tangney et al. (2004), simplified by Morean et al. (2014), and translated into Chinese by Luo et al. (2021). This version consists of seven items that assess two dimensions: self-discipline (e.g., “I am good at resisting temptations”) and impulse control (e.g., “I do certain things that are bad for me, if they are fun”). The Brief Self-Control Scale utilizes a five-point scoring system to measure individuals’ abilities in behavioral regulation, with higher scores indicating better self-control. The self-discipline subscale demonstrated a Cronbach’s alpha of 0.64, while the impulse control subscale, 0.68. Because these scores were slightly low, we ran a Confirmatory Factor Analysis (CFA) to check the scale’s validity. The results showed a good fit (*χ^2^*/df = 1.697, CFI = 0.978, TLI = 0.965, RMSEA = 0.076), demonstrating that the scale possessed adequate construct validity for the present study.

#### 2.2.4 The social emotional learning ability scale

The Social Emotional Learning Ability scale (student version) was developed by Zheng (2017), drawing on the social and emotional learning standards established in Illinois, United States. The scale assesses social emotional skills in late elementary students (ages 9–11) using three sub-scales and 10 dimensions, totaling 58 items. These items encompass self-awareness and self-management, social cognition and interpersonal communication, decision-making skills, and responsible behavior. For this study, the sub-scales on self-awareness (e.g., “I am able to detect and reflect on changes in my emotions at any time”) and self-management (e.g., “I can adjust the pace in a timely manner according to the degree of achievement of the plan”) were selected, as emotional management is a crucial developmental skill in social emotional skills. Participants responded using a five-point Likert scale (0 = strongly disagree, 4 = strongly agree). The Cronbach’s alpha for self-awareness and self-management were 0.85 and 0.81, respectively.

### 2.3 Procedure

The study procedures were conducted in accordance with the Declaration of Helsinki, and ethical approval (no.: 20211207) was granted by the Ethics Review Committee of Huzhou University, China. Because the data collection was integrated into the schools’ mental health education program, overarching consent was provided by school administrators on behalf of the students’ families. Nevertheless, all students and their legal guardians were fully informed about the study’s purpose, and participation was entirely voluntary with the option to withdraw at any time. Data were collected offline during regular school hours. Prior to completing the questionnaires, participants were briefed on the research objectives and assured of strict confidentiality.

To protect student privacy, all responses were submitted anonymously, and the data remained accessible solely to the research team for academic purposes.

### 2.4 Data analysis

The data were analyzed using SPSS 29.0 and PROCESS macro (Version 4.0). Our analysis followed three main steps.

First, to examine the potential influence of common method bias (Common Method Bias, CMB), the unmeasured latent method construct (ULMC) approach was employed (Podsakoff et al., 2003). Specifically, a common method factor (CM) was added to the two-factor measurement model (GQ and CQ), with CM specified to load on all observed indicators. The method factor was constrained to be orthogonal to the substantive latent variables to ensure conceptual independence.

The baseline measurement model without the method factor demonstrated the following fit indices: *χ*^2^ (298) = 1796.14, CFI = 0.760, TLI = 0.738, and RMSEA = 0.0966. After incorporating the common method factor, model fit improved to *χ*^2^(297) = 1572.31, CFI = 0.796, TLI = 0.777, and RMSEA = 0.0893 (ΔCFI = 0.036; ΔRMSEA = −0.0073). Further analyses indicated that the common method factor accounted for an average of approximately 7.49% of the variance across indicators (range = 7.01–7.81%), which is substantially below the commonly suggested threshold of concern (25%). These results suggest that common method bias is unlikely to pose a serious threat to the validity of the study’s conclusions.

We then ran descriptive statistics and correlations to examine relationships between variables. Second, we used Analysis of Variance (ANOVA) to identify any differences based on student ability and family status. Variables that affected the results were then treated as controls (covariates) in the final model.

Third, to test our hypotheses, we built a chain mediation model. We chose Hayes’ Model 6 because our plan involved two mediating variables working in a sequence. We used bootstrapping (5,000 samples) to make sure the indirect paths were significant. We also controlled for sex, grade, ability, and family background (Borgonovi and Han, 2021) to ensure accuracy.

## 3 Results

### 3.1 Preliminary analysis of covariates

The results indicated significant differences between self-reported ability levels: growth mindset [*F* (5,532) = 10.96, *p* < 0.001], negative automatic thoughts [*F* (5,532) = 26.75, *p* < 0.001], self-control [*F* (5,532) = 38.07, *p* < 0.001], and social emotional skills [*F* (5,532) = 50.47, *p* < 0.001]. Similarly, there were significant differences between different socioeconomic status levels: growth mindset [*F* (4,533) = 6.66, *p* < 0.001], negative automatic thoughts [*F* (4,533) = 3.18, *p* < 0.001], self-control [*F* (4,533) = 6.36, *p* < 0.001], and social emotional skills [*F* (4,533) = 7.90, *p* < 0.001] (Tables 1, 2). Because ability and social economic status affected the main variables, we treated them as control variables (covariates) in all later regression and mediation analyses to ensure our findings were accurate.

**Table 1:** Demographic variable analysis of ability.

Scale	*M*weak	*M*average	*M*strong	*F*
Growth mindset	3.69	3.94	4.19	10.96^***^
Negative automatic	2.03	1.49	1.52	26.75^***^
Thoughts Self-control	2.82	3.24	3.47	38.07^***^
Social emotional skills	3.32	3.83	4.18	50.47^***^

***p < 0.001.

**Table 2:** Demographic variable analysis of socioeconomic status (SES).

Scale	*M*low	*M*medium	*M*high	*F*
Growth mindset	3.80	4.03	4.28	6.66^***^
Negative automatic	1.72	1.58	1.47	3.18^***^
thoughts self-control	3.11	3.24	3.47	6.36^***^
Social emotional skills	3.68	3.88	4.10	7.90^***^

***p < 0.001.

### 3.2 Correlation analysis

The Pearson correlation analysis (Table 3) revealed significant positive associations between growth mindset and both self-control (*r* = 0.36, *p* < 0.01) and social emotional skills (*r* = 0.36, *p* < 0.01). Additionally, self-control and social emotional skills exhibited a significant positive correlation (*r* = 0.59, p < 0.01). In contrast, significant negative correlations were found between negative automatic thoughts and each of the three variables: growth mindset (*r* = −0.24, *p* < 0.01), self- control (*r* = −0.47, *p* < 0.01), and social emotional skills (*r* = −0.40, *p* < 0.01).

**Table 3:** Correlation analysis for the sample.

Scale	*M ± SD*	1	2	3
Growth mindset	3.96 ± 0.94			
Negative automatic thoughts	1.63 ± 0.75	−0.24^**^		
Self-control	3.20 ± 0.68	0.36^**^	−0.47^**^	
Social emotional skills	3.82 ± 0.79	0.36^**^	−0.40^**^	0.59^**^

1 = Growth mindset; 2 = Negative automatic thoughts; 3 = Self-control. **p < 0.01.

### 3.3 Mediation by negative automatic thoughts and self-control

**Figure 1:**
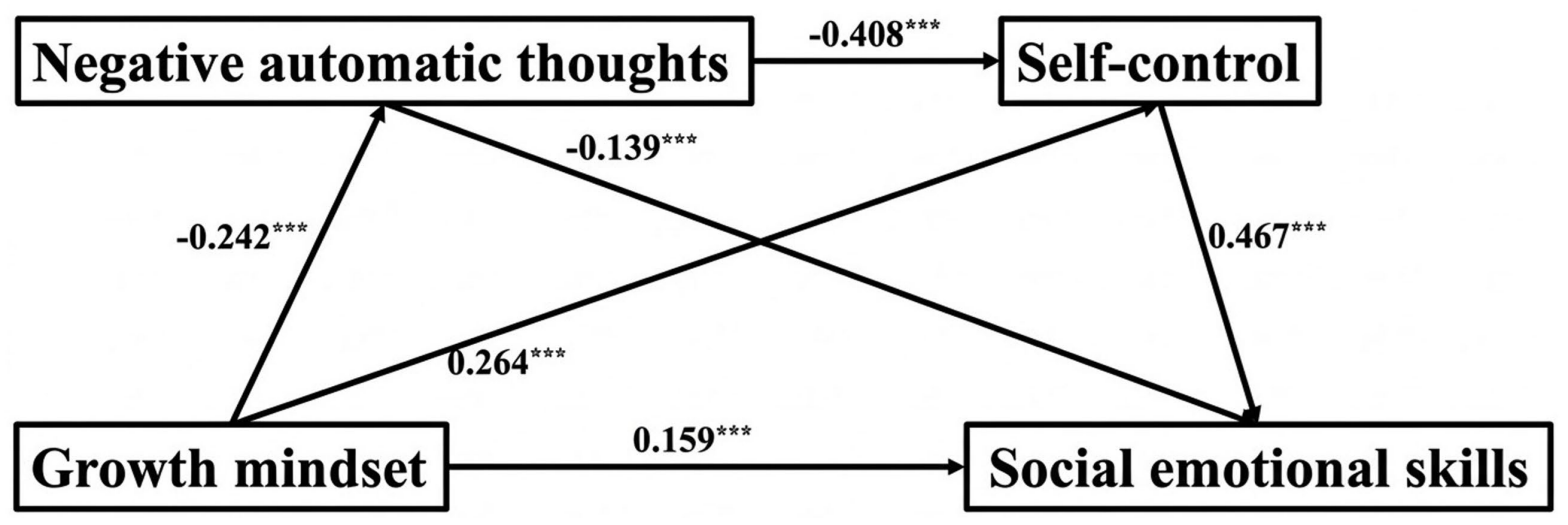
Mediation model for the total sample.

The regression analysis provided empirical support for hypotheses 1 and 4, demonstrating that growth mindset was negatively associated with negative automatic thoughts and positively associated with self-control (*β* = −0.24, *p* < 0.001) and a direct positive association with self-control (*β* = 0.26, *p* < 0.001). Additionally, negative automatic thoughts were negatively associated with self-control (*β* = −0.41, *p* < 0.001). When examined simultaneously, growth mindset and self-control were positively associated with intrapersonal dimensions of social emotional skills, whereas negative automatic thoughts were negatively associated with intrapersonal dimensions of social emotional skills, both growth mindset (*β* = 0.16, *p* < 0.001) and self-control (*β* = 0.47, *p* < 0.001) exhibited significant positive associations, while negative automatic thoughts showed significant negative associations (*β* = −0.14, *p* < 0.001) (Figure 1 and Table 4).

**Table 4:** Mediation analysis findings.

Outcome variable	Model 1		Model 2		Model 3	
	Negative automatic thoughts		Self-control		Social emotional skills	
	*β*	*t*	*β*	*t*	*β*	*t*
Growth mindset	−0.24^***^	−5.80	0.26^***^	7.03	0.16^***^	4.38
Negative automatic thoughts			−0.41^***^	−10.86	−0.14^***^	−3.62
Self-control					0.47^***^	11.67
R^2^	0.06		0.29		0.39	
F	33.59		108.6		113.61	

***p < 0.001.

Mediation analysis (Table 5) showed a total indirect association of 0.20 between growth mindset and the intrapersonal dimensions of social emotional skills, comprising three statistical pathways. This indirect estimate comprised three pathways: (1) growth mindset to negative automatic thoughts to social emotional skills, with a mediation association of 0.03, accounting for 9.4% of the total estimate; (2) growth mindset to self- control to social emotional skills, 0.12, 33.98%; and (3) growth mindset to negative automatic thoughts to self-control to social emotional skills, 0.05, 12.7%. The bootstrap 95% confidence intervals for all three indirect estimates did not include 0, indicating statistical significance. These findings were consistent with hypotheses 2, 3, and 5; the direct association from growth mindset to social emotional skills was the largest, and the indirect path via self-control was the largest among indirect paths.

**Table 5:** All path association analysis.

Model path	*β*	SE	95%CI	Percentage
Total estimate	0.37	0.03	0.24–0.37	
Direct estimate	0.17	0.03	0.07–0.19	43.92%
Indirect estimate	0.20	0.03	0.15–0.26	56.08%
Path 1: Via Neg. Auto.Thoughts	0.03	0.01	0.01–0.06	9.4%
Thought Path 2: Via Self-Control	0.12	0.02	0.08–0.17	33.98%
Path 3: Via Chain (NAT - SC)	0.05	0.01	0.03–0.07	12.7%

### 3.4 Growth mindset versus indifferent mindset

**Figure 2:**
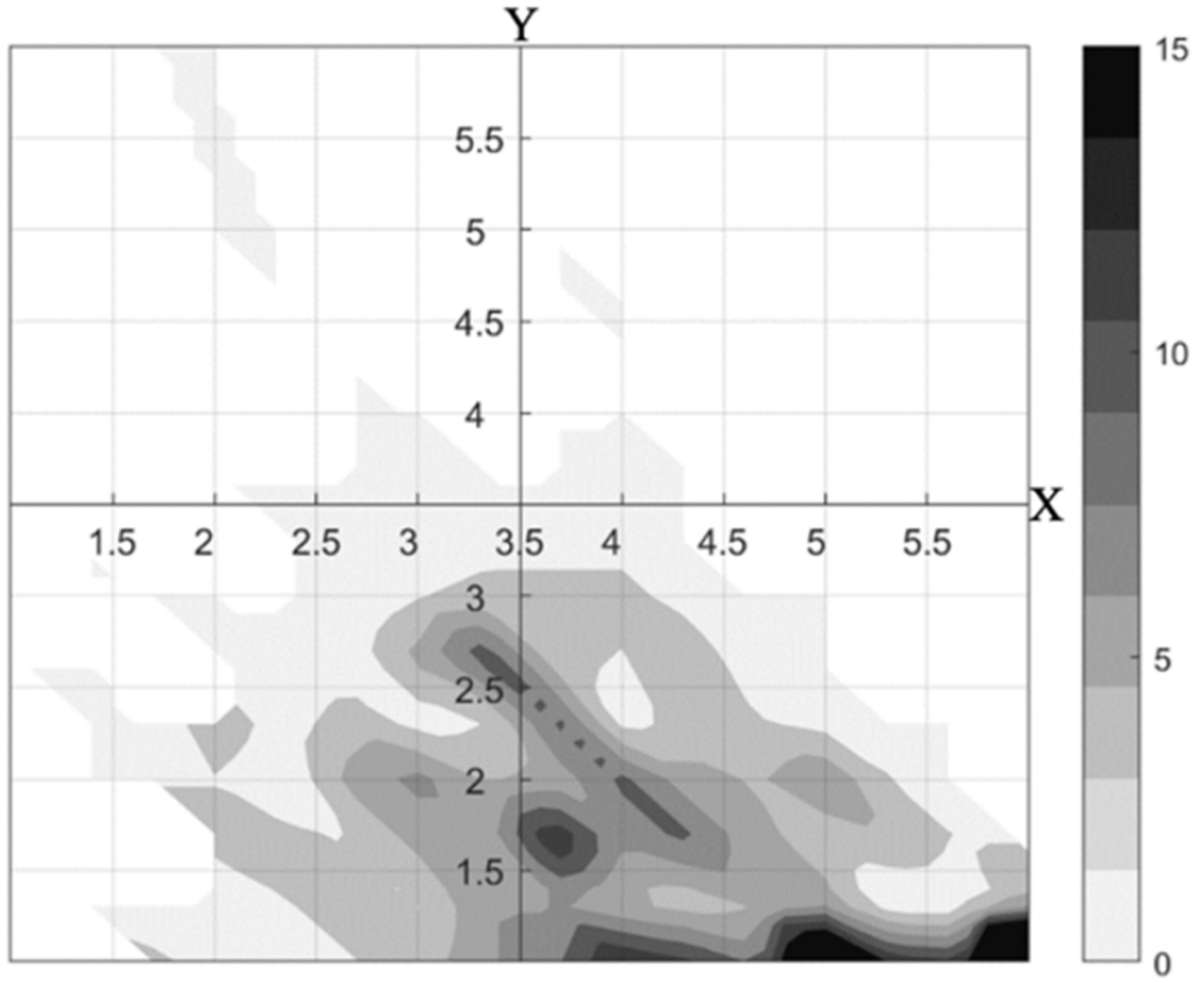
Distribution of mindsets for the total sample (X = growth mindset; Y = fixed mindset).

To further explore how growth mindset relates to students’ social emotional skills, self-control, and negative automatic thoughts, we created a refined classification of mindset patterns using theoretical mean values in Matlab. Four mindset patterns emerged: ambivalent (high growth and high fixed), growth (high growth and low fixed), fixed (low growth and high fixed), and indifferent (low growth and low fixed) (Figure 2).

In the subsequent analysis, we compared growth (*N =* 320) and indifferent mindset patterns (*N =* 165) due to the limited number of participants with ambivalent and fixed mindsets. Both patterns exhibited a low level of fixed mindset but different levels of growth mindset, which was higher in growth mindset than in indifferent mindset.

**Figure 3:**
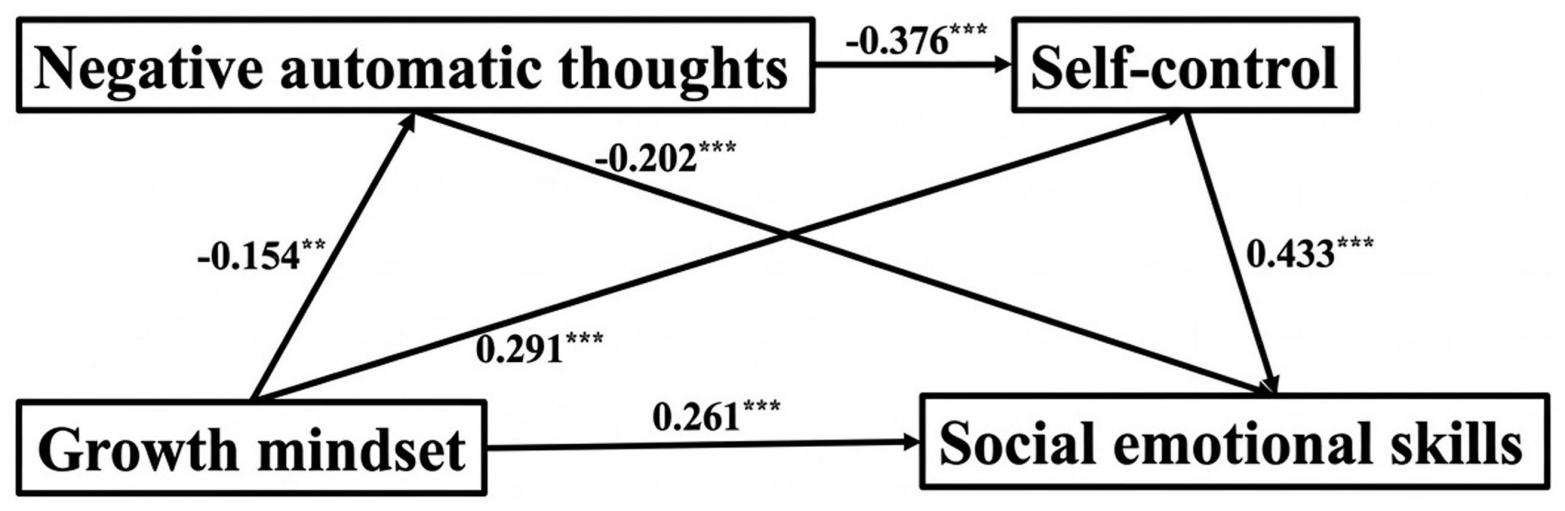
Mediation model for the growth mindset group.

Comparisons suggested lower negative automatic thoughts in the growth mindset group (*β* = −0.15, *p* < 0.01) while also showing a stronger positive correlation with self- control and the intrapersonal dimensions of social emotional skills (*β*total = 0.26, 0.16, *p* < 0.001; *β*growth = 0.29, 0.26, *p* < 0.001). Interestingly, negative automatic thoughts still associated with self-control (*β* = −0.38, *p* < 0.01). The significant relationship between self-control and social emotional skills was also established (*β* = 0.43, *p* < 0.001). Moreover, associations between mindset patterns and social emotional skills were observed in direct, indirect, and total estimates (Figure 3 and Tables 6, 7).

**Table 6:** Mediation analysis for growth mindset.

Outcome variable	Model 1		Model 2		Model 3	
	Negative automatic thoughts		Self-control		Social emotional skills	
	*β*	*t*	*β*	*t*	*β*	*t*
Growth mindset	−0.15^**^	−2.77	0.26^***^	5.94	0.26^***^	5.95
Negative automatic thoughts			−0.38^***^	−7.69	−0.20^***^	−4.45
Self-control					0.47^***^	11.67
R^2^	0.02		0.3		0.47	
*F*	7.69		55.60		91.55	

***p < 0.001.

**Table 7:** All path association analysis for growth mindset.

Model path	*β*	SE	95%CI	Percentage
Total estimate	0.44	0.06	0.41–0.64	
Direct estimate	0.26	0.05	0.21–0.41	58.92%
Indirect estimate	0.18	0.03	0.12–0.25	41.08%
Path 1: Via Neg. Auto.Thoughts	0.031	0.02	0.01–0.06	7%
Path 2: Via Self-Control	0.13	0.03	0.08–0.18	28.44%
Path 3: Via Chain (NAT-SC)	0.03	0.01	0.04–0.08	5.64%

The analysis (Table 7) showed a total association estimate of 0.44 for growth mindset with the intrapersonal dimensions of social emotional skills. This association comprised a direct and indirect estimate of 0.26 and 0.18, respectively, accounting for 41.08% of the total estimate. The indirect estimate was divided into three paths: path 1 = 0.03, representing 7% of the total estimate; path 2 = 0.13, 28.44%; and path 3 = 0.03, 5.64%. Notably, the path via self-control had the largest indirect estimate.

**Figure 4:**
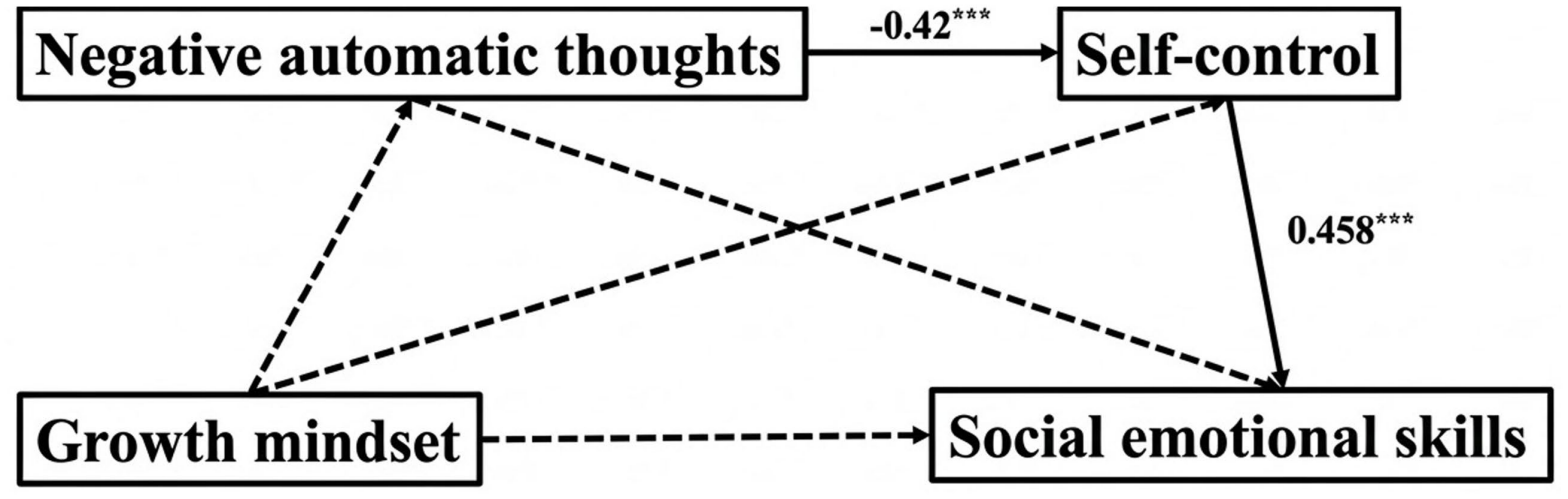
Mediation model for the indifferent mindset group.

A chain mediation model among negative automatic thoughts, self-control, and social emotional skills in students with indifferent mindset (Figure 4 and Table 8).

**Table 8:** Complete mediation analysis for indifferent mindset.

Predictor	Model 1		Model 2		Model 3	
	Negative automatic thoughts		Self-control		Social emotional skills	
	*β*	*T*	*β*	*T*	*β*	*T*
Growth mindset	−0.11	−1.47	0.06	0.90	0.03	0.38
Negative automatic thoughts			−0.42^***^	−5.87	−0.13	−1.67
Self-control					0.46^***^	6.16
R^2^	0.57				0.28	
*F*	9.81		19.24		20.62	

***p < 0.001.

## 4 Discussion

This research aimed to explore the relationship between growth mindset, negative automatic thoughts, self-control, and the intrapersonal dimensions of social emotional skills in fifth and sixth grade students. Using a serial mediation model, the current study demonstrated that a growth mindset positively predicts the intrapersonal dimensions of social emotional skills, both directly and indirectly through reduced negative automatic thoughts and enhanced self-control.

Additionally, the study introduced the concept of indifferent mindset and compared it with growth mindset. Individuals classified with a growth mindset showed higher self-control and the intrapersonal dimensions of social emotional skills scores. Unlike the growth mindset group, the indifferent mindset group did not exhibit significantly lower levels of negative automatic thoughts.

### 4.1 Relationships among growth mindset, negative automatic thoughts, and social emotional skills

This study found that negative automatic thoughts play a crucial role in the relationship between mindset patterns, self-control, and social emotional skills. Two distinct mediation paths were identified. Negative automatic thoughts were negatively associated with social emotional skills in all participants and in those with growth mindset and indirectly associated with social emotional skills through self-control abilities. Notably, only the indirect pathway connecting negative automatic thoughts and social emotional skills was observed in the indifferent mindset group.

Mindset patterns are associated with negative automatic thoughts. Previous research has shown that these patterns can decrease stress (Yeager et al., 2016), anxiety, and depression (Schleider and Weisz, 2018), while improving subjective wellbeing (Ortiz Alvarado et al., 2019). Our findings align with Beck’s cognitive-behavioral theory, suggesting that growth mindset revolves around the belief that one’s intelligence, abilities, and emotions are malleable. This mindset has been hypothesized to be related to stress responses and is associated with lower negative automatic thoughts and reduced anxiety in some studies (Crum et al., 2017).

The emergence of negative automatic thoughts may be related to by various factors, including environmental stressors and an individual’s stress response, known as stress mindset (Crum et al., 2013). Research suggests that stress can impede personal development and is linked to problems such as psychological anxiety, emotional disorders, anorexia, cardiovascular diseases (Muris et al., 2009). While interventions typically aim to mitigate the negative association of stress, it is crucial to recognize that stress is often unavoidable and inherent to development. Instead, stressors can present opportunities for individuals to respond positively. Therefore, one should focus on optimizing their stress responses and view stress as a tool for self-improvement (Crum et al., 2020). A growth mindset fosters persistence, learning-oriented goals, and mastery-oriented strategies, motivating individuals to solve problems independently or seek appropriate assistance (Doron et al., 2009; Dweck et al., 1995; Yeager and Dweck, 2012).

Negative automatic thoughts are associated with by an individual’s personality traits and attribution style. According to Eysenck’s dual personality theory and Abramson’s attribution theory, extroverted individuals tend to optimistically attribute positive outcomes, while those with neurosis tend to have a pessimistic attribution style for negative events. People with growth mindset show more resilience in response to failure and frustration, opting for rational attributions rather than labeling themselves as unintelligent.

### 4.2 Relationships among growth mindset, self-control, and social emotional skills

Self-control consistently mediated the relationship between growth mindset and social emotional skills, although this pathway was non-significant within the indifferent mindset group. Previous research has shown that higher levels of self-control are associated with improved academic achievement, adaptability, self-esteem, relationships, interpersonal skills, and emotional responses (Tangney et al., 2004).

The growth mindset theory suggests that abilities can be developed through effort, potentially fostering resilience (Wang et al., 2018). Embracing growth mindset may help mitigate self-sabotage and emotional discord, resist temptation, and regulate emotions (Bernecker and Job, 2017; Konze et al., 2019). Our study supports Burnette’s findings, indicating that growth mindset is positively associated with self-regulation abilities, including behavioral adjustment and self-control (Burnette et al., 2013). Further research on subcomponents such as self-esteem, self-control, and self-efficacy within growth mindset may help develop more effective interventions.

For fifth and sixth-grade students beginning their academic journey, current technological advancements highlight the significance of continuous learning. Education is a long-term developmental process analogous to a marathon, requiring sustained effort and self-regulation. In embracing growth mindset as a core belief, self-discipline becomes crucial (Ku and Stager, 2022), serving as a “bridge” linking to social emotional skills, self-awareness, and the capacity to adjust to societal shifts.

### 4.3 The mediation path from growth mindset to the intrapersonal dimensions of social emotional skills

Our findings identified three key mediating pathways linking growth mindset to social emotional skills, thereby providing empirical support for an integrative framework that combines the Cognitive Model and Self-Regulation Theories. Specifically, growth mindset exerted its influence through (1) negative automatic thoughts, (2) self-control, and (3) a serial pathway in which cognitive processes preceded regulatory mechanisms. This finding corroborates the meta-analytic evidence reported by Burnette et al. (2013), suggesting that the primary functional value of a growth mindset lies in its capacity to sustain self-regulatory processes, rather than merely to modify cognitive content. The central role of self-control observed in our model corroborates previous research by Tangney et al. (2004), confirming its importance in behavioral regulation.

Moreover, although growth mindset was associated with significantly lower levels of negative automatic thoughts—aligning with its protective role against psychopathology (Schleider et al., 2015)—the pathway operating solely through negative automatic thoughts exhibited the weakest association with social emotional skills. This pattern suggests that adaptive behavior is most effectively promoted when cognitive changes, such as reductions in negative automatic thoughts, are successfully translated into behavioral regulation via self-control. Such a cognition-to-regulation process is central to the development of resilience, as emphasized by Yeager and Dweck (2012).

### 4.4 Relationships between growth mindset and indifferent mindset

Research on participants with different mindsets, such as growth and indifferent, consistently shows the importance of mindset patterns in social emotional skills. The study also highlights the significance of negative automatic thoughts and self-control in this relationship. Furthermore, Dweck et al. (1995) recognized that some individuals may hold both growth and fixed mindsets simultaneously, a concept referred to as the “ambivalent mindset” by Chiu et al. (2023). Similarly, our research identified a mindset that is indifferent to both growth and fixed patterns, known as the “indifferent mindset.”

The findings indicated that individuals with a pure growth mindset were associated with stronger protective and buffering associations of mindset patterns and exhibited higher levels of self-control and social emotional skills than other groups. In contrast, indifferent mindset showed no significant positive association with individual development. Individuals with an indifferent mindset fail to derive the protective benefits of a growth mindset, consequently exhibiting poorer self-control, lower social emotional skills, and more frequent negative automatic thought. Thus, for students with an indifferent mindset, their beliefs about intelligence seem disconnected from their negative thoughts. Unlike the growth mindset, which helps reduce negative thinking, the indifferent mindset offers no such protection. Consequently, negative cognitions may exert a more detrimental impact on self-control and social emotional skills in the absence of the buffering effect of a growth mindset.

### 4.5 Educational implication

This study shows that growth mindset links to social emotional skills mostly through self-control. This suggests that interventions solely focused on instilling the belief in malleability may be insufficient. Schools should also focus on enhancing self- control and reducing negative thoughts to foster social skills. Furthermore, particular attention should be paid to the “indifferent mindset” group identified in our study. These students seem disconnected from their belief systems, leaving them without the protection that a growth mindset usually offers. Therefore, teachers need to pay special attention to these indifferent mindset students. These students may require tailored interventions designed to foster emotional engagement prior to addressing their mindset beliefs.

### 4.6 Limitations of the current study

Due to the limited number of participants in fixed and ambivalent mindset groups, the data from only the growth and indifferent mindset groups were considered for the analysis. Data from the indifferent mindset group showed no correlation between mindset and negative automatic thoughts, self-control, and social emotional skills. This suggests that participants’ indifference towards the malleability of their own intelligence may be a factor in this outcome. However, it is important to acknowledge that other factors may have influenced the results. First, the experimenters did not adequately guide participants in understanding the concepts of each mindset pattern, potentially affecting the objectivity of the data collected. Second, traditional measures of growth mindset primarily assess mindset patterns in middle and high school students, which may not fully capture the cognitive understanding of intelligence in elementary school students (Kuhfeld et al., 2023). Additionally, elementary school students’ ideal self-concept may relate to their responses to the growth mindset scale, potentially resulting in data that is not entirely objective or truthful. Furthermore, the study on elementary school students had a limited number of participants and only utilized cross- sectional studies, which is a significant research limitation.

Because implicit theories are domain-specific (Schroder et al., 2016), measuring mindsets primarily regarding intelligence may underestimate their true association with the emotional domains of social emotional skills. Due to the limited number of studies exploring mindset categorization, the current research took an exploratory approach. Mindsets were categorized based on theoretical averages across different dimensions, with the rationality of these criteria still being debated. For instance, According to Chiu et al. (2023), an ambivalent mindset was defined as cases in which scores on both the fixed mindset and growth mindset dimensions exceeded 5. The categorization of mindset patterns in the study may be scientifically justified or incorrect; hence, future research should involve more scholars to further explore and critique mindset classification and provide suggestions for improvement.

Third, by focusing solely on intrapersonal dimensions (self-management and self-awareness), our findings reflect internal self-regulatory competence rather than the broader interpersonal social functioning defined by the full CASEL framework.

## 5 Conclusion

This study found that growth mindset was directly associated with the intrapersonal dimensions of social emotional skills and showed indirect associations via negative automatic thoughts and self-control. The largest indirect association linking growth mindset and social emotional skills was via self-control. Furthermore, participants with specific mindsets showed distinct associations with and pathways between negative automatic thoughts, self-control, and social emotional skills. Individuals with growth mindset had higher self-control and social emotional skills scores; those with indifferent mindset showed weaker associations with social emotional skills.

## Data Availability

The raw data supporting the conclusions of this article will be made available by the authors, without undue reservation.

## References

[ref1] Aronson J. Fried C. B. Good C. (2002). Reducing the effects of stereotype threat on African American college students by shaping theories of intelligence. J. Exp. Soc. Psychol. 38, 113–125. doi: 10.1006/jesp.2001.1491

[ref2] Beck A. T. (1976). Cognitive Therapy and the Emotional Disorders. New York, NY: International Universities Press.

[ref3] Beck J. S. (2021). Cognitive Behavior Therapy: Basics and beyond. London: The Guilford Press.

[ref4] Bernecker K. Job V. (2017). “Implicit theories about willpower and their implications for health and well-being,” in Routledge International Handbook of Self-Control in Health and well-Being, eds. Ridr D. Adriaanse M. Fujita K. (London: Routledge), 143–155.

[ref5] Blackwell L. S. Trzesniewski K. H. Dweck C. S. (2007). Implicit theories of intelligence predict achievement across an adolescent transition: a longitudinal study and an intervention. Child Dev. 78, 246–263. doi: 10.1111/j.1467-8624.2007.00995.x, 17328703

[ref6] Borgonovi F. Han S. W. (2021). Gender disparities in fear of failure among 15- year-old students: the role of gender inequality, the organisation of schooling and economic conditions. J. Adolesc. 86, 28–39. doi: 10.1016/j.adolescence.2020.11.009, 33302248

[ref7] Burnette J. L. Babij A. D. Oddo L. E. Knouse L. E. (2020a). Self-regulation mindsets: relationship to coping, executive functioning, and ADHD. J. Soc. Clin. Psychol. 39, 101–116. doi: 10.1521/jscp.2020.39.02.101

[ref8] Burnette J. L. Knouse L. E. Vavra D. T. O’Boyle E. Brooks M. A. (2020b). Growth mindsets and psychological distress: a meta-analysis. Clin. Psychol. Rev. 77:101816. doi: 10.1016/j.cpr.2020.101816 32163802

[ref9] Burnette J. L. O’Boyle E. H. VanEpps E. M. Pollack J. M. Finkel E. J. (2013). Mind-sets matter: a meta-analytic review of implicit theories and self-regulation. Psychol. Bull. 139, 655–701. doi: 10.1037/a0029531, 22866678

[ref10] Calvete E. Orue I. Hankin B. L. (2013). Early maladaptive schemas and social anxiety in adolescents: the mediating role of anxious automatic thoughts. J. Anxiety Disord. 27, 278–288. doi: 10.1016/j.janxdis.2013.02.011, 23602941

[ref11] Canning E. A. Muenks K. Green D. J. Murphy M. C. (2019). STEM faculty who believe ability is fixed have larger racial achievement gaps and inspire less student motivation in their classes. Sci. Adv. 5:eaau4734. doi: 10.1126/sciadv.aau4734, 30793027 PMC6377274

[ref12] Canning E. A. Ozier E. Williams H. E. AlRasheed R. Murphy M. C. (2022). Professors who signal a fixed mindset about ability undermine women’s performance in STEM. Soc. Psychol. Personal. Sci. 13, 927–937. doi: 10.1177/19485506211030398

[ref13] Carter J. S. Garber J. Ciesla J. A. Cole D. A. (2006). Modeling relations between hassles and internalizing and externalizing symptoms in adolescents: a four-year prospective study. J. Abnorm. Psychol. 115, 428–442. doi: 10.1037/0021-843X.115.3.428, 16866584

[ref14] CASEL. (2023). What is the CASEL Framework? Available online at: https://casel.org/fundamentals-of-sel/what-is-the-casel-framework/ (accessed December 29, 2023).

[ref15] Chapman D. P. Whitfield C. L. Felitti V. J. Dube S. R. Edwards V. J. (2004). Adverse childhood experiences and the risk of depressive disordersi n adulthood. J. Affect. Disord. 82, 217–225. doi: 10.1016/j.jad.2003.12.013, 15488250

[ref16] Chen P. Powers J. T. Katragadda K. R. Cohen G. L. Dweck C. S. (2020). A strategic mindset: an orientation toward strategic behavior during goal pursuit. Proc. Natl. Acad. Sci. USA 117, 14066–14072. doi: 10.1073/pnas.2002529117, 32522882 PMC7322028

[ref17] Chiu C. Y. Tong Y. Y. Lee S. L. Chan H. S. (2023). Personal qualities are malleable and fixed: ambivalent mindset, capability ranking reinforcement, and parent–child relationship among Hong Kong Chinese parents. J. Pac. Rim Psychol. 17:183449092311661. doi: 10.1177/18344909231166106

[ref18] Choon M. W. Talib M. Yaacob S. N. Awang H. Tan J. P. Hassan S. . (2015). Negative automatic thoughts as a mediator of the relationship between depression and suicidal behaviour in an at-risk sample of Malaysian adolescents. Child Adolesc. Ment. Health 20, 89–93. doi: 10.1111/camh.12075 32680393

[ref19] Cipriano C. Strambler M. J. Naples L. H. Ha C. Kirk M. Wood M. . (2023). The state of evidence for social and emotional learning: a contemporary meta-analysis of universal school-based SEL interventions. Child Dev. 94, 1181–1204. doi: 10.1111/cdev.13968, 37448158

[ref20] Clark D. A. Beck A. T. (2010). Cognitive theory and therapy of anxiety and depression: convergence with neurobiological findings. Trends Cogn. Sci. 14, 418–424. doi: 10.1016/j.tics.2010.06.007, 20655801

[ref21] Costa A. Faria L. (2018). Implicit theories of intelligence and academic achievement: a meta-analytic review. Front. Psychol. 9:829. doi: 10.3389/fpsyg.2018.00829, 29922195 PMC5996155

[ref22] Crandell C. J. Chambless D. L. (1986). The validation of an inventory for measuring depressive thoughts: the Crandell cognitions inventory. Behav. Res. Ther. 24, 403–411. doi: 10.1016/0005-7967(86)90005-7, 3741306

[ref23] Crum A. J. Akinola M. Martin A. Fath S. (2017). The role of stress mindset in shaping cognitive, emotional, and physiological responses to challenging and threatening stress. Anxiety Stress Coping 30, 379–395. doi: 10.1080/10615806.2016.1275585, 28120622

[ref24] Crum A. J. Jamieson J. P. Akinola M. (2020). Optimizing stress: an integrated intervention for regulating stress responses. Emotion 20, 120–125. doi: 10.1037/emo0000670, 31961190 PMC7608610

[ref25] Crum A. J. Salovey P. Achor S. (2013). Rethinking stress: the role of mindsets in determining the stress response. J. Pers. Soc. Psychol. 104, 716–733. doi: 10.1037/a0031201, 23437923

[ref26] Davidson R. J. Begley S. (2012). The Emotional life of your brain: How its Unique Patterns affect the way you Think, Feel, and live, and how you can change them. New York, NY: Hudson Street Press.

[ref27] Domitrovich C. E. Durlak J. A. Staley K. C. Weissberg R. P. (2017). Social- emotional competence: an essential factor for promoting positive adjustment and reducing risk in school children. Child Dev. 88, 408–416. doi: 10.1111/cdev.12739, 28213889

[ref28] Doron J. Stephan Y. Boiché J. Scanff C. L. (2009). Coping with examinations: exploring relationships between students' coping strategies, implicit theories of ability, and perceived control. Br. J. Educ. Psychol. 79, 515–528. doi: 10.1348/978185409X402580, 19187577

[ref29] Duckworth A. Gross J. J. (2014). Self-control and grit: related but separable determinants of success. Curr. Dir. Psychol. Sci. 23, 319–325. doi: 10.1177/0963721414541462, 26855479 PMC4737958

[ref30] Duckworth A. L. Seligman M. E. P. (2005). Self-discipline outdoes IQ in predicting academic performance of adolescents. Psychol. Sci. 16, 939–944. doi: 10.1111/j.1467-9280.2005.01641.x, 16313657

[ref31] Duckworth A. L. Taxer J. L. Eskreis-Winkler L. Galla B. M. Gross J. J. (2019). Self-control and academic achievement. Annu. Rev. Psychol. 70, 373–399. doi: 10.1146/annurev-psych-010418-103230, 30609915

[ref32] Durlak J. A. Weissberg R. P. Dymnicki A. B. Taylor R. D. Schellinger K. B. (2011). The impact of enhancing students’ social and emotional learning: a meta-analysis of school-based universal interventions. Child Dev. 82, 405–432. doi: 10.1111/j.1467-8624.2010.01564.x, 21291449

[ref33] Dweck C. S. (1999). Self-Theories: Their role in Motivation, Personality, and Development. London: Psychology Press.

[ref34] Dweck C. S. (2006). Mindset: The new Psychology of success. London: Random House.

[ref35] Dweck C. (2012). Mindset: How you can Fulfil your Potential. London: Robinson.

[ref36] Dweck C. S. Chiu C. Hong Y. (1995). Implicit theories: elaboration and extension of the model. Psychol. Inq. 6, 322–333. doi: 10.1207/s15327965pli0604_12

[ref37] Dweck C. S. Leggett E. L. (1988). A social-cognitive approach to motivation and personality. Psychol. Rev. 95, 256–273. doi: 10.1037/0033-295X.95.2.256

[ref38] Elias M. Arnold H. (2006). The educator’s guide to emotional intelligence and academic achievement: social-emotional learning in the classroom. Available online at: https://www.semanticscholar.org/paper/The-educator’s-guide-to-emotional-intelligence-and-Elias-Arnold/5b863b830c80d606b3daa634785dbbd1f7792467

[ref39] Elias M. J. Zins J. E. Weissberg R. P. Frey K. S. Greenberg M. T. Haynes N. M. . (1997). Promoting social and Emotional Learning: Guidelines for Educators. Cham: Springer.

[ref40] Flouri E. Panourgia C. (2014). Negative automatic thoughts and emotional and behavioural problems in adolescence. Child Adolesc. Mental Health 19, 46–51. doi: 10.1111/camh.12004, 32878364

[ref41] Greenberg M. T. Weissberg R. P. O’Brien M. U. Zins J. E. Fredericks L. Resnik H. . (2003). Enhancing school-based prevention and youth development through coordinated social, emotional, and academic learning. Am. Psychol. 58, 466–474. doi: 10.1037/0003-066X.58.6-7.466, 12971193

[ref42] Gross J. J. John O. P. (2003). Individual differences in two emotion regulation processes: implications for affect, relationships, and well-being. J. Pers. Soc. Psychol. 85, 348–362. doi: 10.1037/0022-3514.85.2.348 12916575

[ref43] Hawkins J. D. Smith B. H. Catalano R. F. (2004). “Social development and social and emotional learning,” in Building academic success on social and Emotional Learning: What does the Research say? eds. Zins J. E. Weissberg R. P. Wang M. C. Walberg H. J. (London: Teachers College Press), 135–150.

[ref44] Hofmann W. Schmeichel B. J. Baddeley A. D. (2012). Executive functions and self-regulation. Trends Cogn. Sci. 16, 174–180. doi: 10.1016/j.tics.2012.01.006 22336729

[ref45] Hollon S. D. Kendall P. C. (1980). Cognitive self-statements in depression: development of an automatic thoughts questionnaire. Cogn. Ther. Res. 4, 383–395. doi: 10.1007/BF01178214

[ref46] Huang C. (2015). Academic achievement and subsequent depression: a meta-analysis of longitudinal studies. J. Child Fam. Stud. 24, 434–442. doi: 10.1007/s10826-013-9855-6

[ref47] Huang Z. Shang K. Zhang J. (2023). Does growth mindset affect students’ social and emotional skills development: empirical analyses based on OECD social and emotional skills study. J. East China Norm. Univ. 41, 22–32. doi: 10.16382/j.cnki.1000-5560.2023.04.002

[ref48] Iancu I. Bodner E. Joubran S. Lupinsky Y. Barenboim D. (2015). Negative and positive automatic thoughts in social anxiety disorder. Isr. J. Psychiatry Relat. Sci. 52, 129–135, 26431418

[ref49] Jiang X. Mueller C. E. Paley N. (2024). A systematic review of growth mindset interventions targeting youth social–emotional outcomes. Sch. Psychol. Rev. 53, 251–272. doi: 10.1080/2372966X.2022.2151321

[ref50] Jones D. E. Greenberg M. Crowley M. (2015). Early social-emotional functioning and public health: the relationship between kindergarten social competence and future wellness. Am. J. Public Health 105, 2283–2290. doi: 10.2105/AJPH.2015.302630, 26180975 PMC4605168

[ref51] Knoch D. Nash K. (2015). “Self-control in social decision making: a neurobiological perspective,” in Handbook of Biobehavioral Approaches to Self-Regulation, eds. Gendolla G. Tops M. Koole S. (Cham: Springer).

[ref52] Knouse L. E. Ziegler M. Lavine I. Zhang J. Cheng Y. Ul Ain H. (2023). Avoidant automatic thoughts are associated with task avoidance and inattention in the moment. Cogn. Ther. Res. 48, 866–879. doi: 10.1007/s10608-023-10410-8

[ref53] Konze A.-K. Rivkin W. Schmidt K.-H. (2019). Can faith move mountains? How implicit theories about willpower moderate the adverse effect of daily emotional dissonance on ego-depletion at work and its spillover to the home- domain. Eur. J. Work Organ. Psy. 28, 137–149. doi: 10.1080/1359432X.2018.1560269

[ref54] Ku Y.-R. Stager C. (2022). Rethinking the multidimensionality of growth mindset amid the COVID-19 pandemic: a systematic review and framework proposal. Front. Psychol. 13:572220. doi: 10.3389/fpsyg.2022.572220 35846666 PMC9284032

[ref55] Kuhfeld M. Soland J. Lewis K. (2023). Investigating differences in how parents and teachers rate students' self-control. Psychol. Assess. 35, 23–31. doi: 10.1037/pas0001187, 36355691

[ref56] Lemerise E. A. Arsenio W. F. (2000). An integrated model of emotion processes and cognition in social information processing. Child Dev. 71, 107–118. doi: 10.1111/1467-8624.00124 10836564

[ref57] Leung P. W. L. Poon M. W. L. (2001). Dysfunctional schemas and cognitive distortions in psychopathology: a test of the specificity hypothesis. J. Child Psychol. Psychiatry 42, 755–765. doi: 10.1111/1469-7610.00772, 11583248

[ref58] Li Y. Bates T. C. (2019). You can’t change your basic ability, but you work at things, and that’s how we get hard things done: testing the role of growth mindset on response to setbacks, educational attainment, and cognitive ability. J. Exp. Psychol. Gen. 148, 1640–1655. doi: 10.1037/xge0000669 31464486

[ref59] Liew J. (2012). Effortful control, executive functions, and education: bringing self- regulatory and social-emotional competencies to the table. Child Dev. Perspect. 6, 105–111. doi: 10.1111/j.1750-8606.2011.00196.x

[ref60] Lin J. (2023). Questionnaire establishment of cognitive bias in depression and case study among senior high school. [Master’s thesis, Guangxi Normal University, China] doi: 10.27036/d.cnki.ggxsu.2023.000614

[ref61] Luo T. Cheng L. M. Qin L. X. Xiao S. Y. (2021). Validation of the Chinese version of the brief self-control scale. Chin. J. Clin. Psych. 29, 83–86. doi: 10.16128/j.cnki.1005-3611.2021.01.017

[ref62] McKown C. Russo-Ponsaran N. M. Allen A. Johnson J. K. Warren-Khot H. K. (2015). Social–emotional factors and academic outcomes among elementary- aged children. Infant Child Dev. 25, 119–136. doi: 10.1002/icd.1926

[ref63] McMahon S. D. Grant K. E. Compas B. E. Thurm A. E. Ey S. (2003). Stress and psychopathology in children and adolescents: is there evidence of specificity? J. Child Psychol. Psychiatry 44, 107–133. doi: 10.1111/1469-7610.00105 12553415

[ref64] Moffitt T. E. Arseneault L. Belsky D. Dickson N. Hancox R. J. Harrington H. . (2011). A gradient of childhood self-control predicts health, wealth, and public safety. Proc. Natl. Acad. Sci. 108, 2693–2698. doi: 10.1073/pnas.1010076108, 21262822 PMC3041102

[ref65] Morean M. E. DeMartini K. S. Leeman R. F. Pearlson G. D. Anticevic A. Krishnan-Sarin S. . (2014). Psychometrically improved, abbreviated versions of three classic measures of impulsivity and self- control. Psychol. Assess. 26, 1003–1020. doi: 10.1037/pas0000003, 24885848 PMC4152397

[ref66] Muris P. Mayer B. den Adel M. Roos T. van Wamelen J. (2009). Predictors of change following cognitive-behavioral treatment of children with anxiety problems: a preliminary investigation on negative automatic thoughts and anxietycontrol. Child Psychiatry Hum. Dev. 40, 139–151. doi: 10.1007/s10578-008-0116-7 18661229 PMC2779362

[ref67] Ortiz Alvarado N. B. Rodríguez Ontiveros M. Ayala Gaytán E. A. (2019). Do mindsets shape students’ well-being and performance? J. Psychol. 153, 843–859. doi: 10.1080/00223980.2019.1631141, 31314699

[ref68] Osher D. Berg J. (2017). School Climate and social and Emotional Learning. University Park, PA: Edna Bennett Pierce Prevention Research Center, The Pennsylvania State University.

[ref69] Podsakoff P. M. MacKenzie S. B. Lee J. Y. Podsakoff N. P. (2003). Common method biases in behavioral research: a critical review of the literature and recommended remedies. J. Appl. Psychol. 88, 879–903. doi: 10.1037/0021-9010.88.5.879 14516251

[ref70] Rissanen I. Laine S. Puusepp I. Kuusisto E. Tirri K. (2021). Implementing and evaluating growth mindset pedagogy—a study of Finnish elementary school teachers. Front. Educ. 6:753698. doi: 10.3389/feduc.2021.753698

[ref71] Savvides H. Bond C. (2021). How does growth mindset inform interventions in primary schools? A systematic literature review. Educ. Psychol. Pract. 37, 134–149. doi: 10.1080/02667363.2021.1879025

[ref72] Schleider J. L. Abel M. R. Weisz J. R. (2015). Implicit theories and youth mental health problems: a random-effects meta-analysis. Clin. Psychol. Rev. 35, 1–9. doi: 10.1016/j.cpr.2014.11.001, 25462109

[ref73] Schleider J. Weisz J. (2018). A single-session growth mindset intervention for adolescent anxiety and depression: 9-month outcomes of a randomized trial. J. Child Psychol. Psychiatry 59, 160–170. doi: 10.1111/jcpp.12811, 28921523

[ref74] Schniering C. A. Lyneham H. J. (2007). The children’s automatic thoughts scale in a clinical sample: psychometric properties and clinical utility. Behav. Res. Ther. 45, 1931–1940. doi: 10.1016/j.brat.2006.09.009, 17078927

[ref75] Schniering C. A. Rapee R. M. (2002). Development and validation of a measure of children’s automatic thoughts: the children’s automatic thoughts scale. Behav. Res. Ther. 40, 1091–1109. doi: 10.1016/S0005-7967(02)00022-0, 12296494

[ref76] Schroder H. S. Dawood S. Yalch M. M. Donnellan M. B. Moser J. S. (2016). Evaluating the domain specificity of mental health–related mind-sets. Soc. Psychol. Personal. Sci. 7, 508–520. doi: 10.1177/1948550616644657

[ref77] Spitzer B. Aronson J. (2015). Minding and mending the gap: social psychological interventions to reduce educational disparities. Br. J. Educ. Psychol. 85, 1–18. doi: 10.1111/bjep.12067 25689030

[ref78] Sun X. Nancekivell S. Gelman S. A. Shah P. (2021). Growth mindset and academic outcomes: a comparison of US and Chinese students. NPJ Sci. Learn. 6:21. doi: 10.1038/s41539-021-00100-z, 34282154 PMC8290023

[ref79] Sun L. Rapee R. M. Tao X. Yan Y. Wang S. Xu W. . (2015). Psychometric properties of the children’s automatic thoughts scale (CATS) in Chinese adolescents. Child Psychiatry Hum. Dev. 46, 600–608. doi: 10.1007/s10578-014-0500-4, 25492243

[ref80] Tamir M. John O. P. Srivastava S. Gross J. J. (2007). Implicit theories of emotion: affective and social outcomes across a major life transition. J. Pers. Soc. Psychol. 92, 731–744. doi: 10.1037/0022-3514.92.4.731, 17469955

[ref81] Tang Y.-Y. Posner M. I. Rothbart M. K. Volkow N. D. (2015). Circuitry of self- control and its role in reducing addiction. Trends Cogn. Sci. 19, 439–444. doi: 10.1016/j.tics.2015.06.007, 26235449

[ref82] Tangney J. P. Baumeister R. F. Boone A. L. (2004). High self-control predicts good adjustment, less pathology, better grades, and interpersonal success. J. Pers. 72, 271–324. doi: 10.1111/j.0022-506.2004.00263.x 15016066

[ref83] Taylor H. E. Larson S. (1999). Social and emotional learning in middle school. The Clearing House: J. Educ. Strateg. Issues Ideas 72, 331–336. doi: 10.1080/00098659909599420

[ref84] Taylor R. D. Oberle E. Durlak J. A. Weissberg R. P. (2017). Promoting positive youth development through school-based social and emotional learning interventions: a Meta-analysis of follow-up effects. Child Dev. 88, 1156–1171. doi: 10.1111/cdev.12864, 28685826

[ref85] Tiet Q. Q. Bird H. R. Davies M. Hoven C. Cohen P. Jensen P. S. (1998). Adverse life events and resilience. J. Am. Acad. Child Adolesc. Psychiatry 37, 1191–1200. doi: 10.1097/00004583-199811000-00020 9808931

[ref86] Timpano K. R. Schmidt N. B. (2013). The relationship between self-control deficits and hoarding: a multimethod investigation across three samples. J. Abnorm. Psychol. 122, 13–25. doi: 10.1037/a0029760, 22924983

[ref87] Wang S. Dai J. Li J. Wang X. Chen T. Yang X. . (2018). Neuroanatomical correlates of grit: growth mindset mediates the association between gray matter structure and trait grit in late adolescence. Hum. Brain Mapp. 39, 1688–1699. doi: 10.1002/hbm.23944 29331059 PMC6866491

[ref88] Watkins E. R. (2008). Constructive and unconstructive repetitive thought. Psychol. Bull. 134, 163–206. doi: 10.1037/0033-2909.134.2.163 18298268 PMC2672052

[ref89] Xu L. Heilbronner S. Schneider V. Dodge E. (2022). Mindset and academic risk. J. Stu. Res. 11:2861. doi: 10.47611/jsrhs.v11i3.2861

[ref90] Yang C. Chan M. K. Ma T. L. (2020). School-wide social emotional learning (SEL) and bullying victimization: moderating role of school climate in elementary, middle, and high schools. J. Sch. Psychol. 82, 49–69. doi: 10.1016/j.jsp.2020.08.002, 32988463

[ref91] Yeager D. S. (2017). Social and emotional learning programs for adolescents. Futur. Child. 27, 73–94. doi: 10.1353/foc.2017.0004

[ref92] Yeager D. S. Dweck C. S. (2012). Mindsets that promote resilience: when students believe that personal characteristics can be developed. Educ. Psychol. 47, 302–314. doi: 10.1080/00461520.2012.722805

[ref93] Yeager D. S. Dweck C. S. (2020). What can be learned from growth mindset controversies? Am. Psychol. 75, 1269–1284. doi: 10.1037/amp0000794, 33382294 PMC8299535

[ref94] Yeager D. S. Hanselman P. Walton G. M. Murray J. S. Crosnoe R. Muller C. . (2019). A national experiment reveals where a growth mindset improves achievement. Nature 573, 364–369. doi: 10.1038/s41586-019-1466-y, 31391586 PMC6786290

[ref95] Yeager D. S. Romero C. Paunesku D. Hulleman C. S. Schneider B. Hinojosa C. . (2016). Using design thinking to improve psychological interventions: the case of the growth mindset during the transition to high school. J. Educ. Psychol. 108, 374–391. doi: 10.1037/edu0000098, 27524832 PMC4981081

[ref96] Yu M. Lv F. Liu Z. Gao D. (2023). How negative automatic thoughts trigger Chinese adolescents’ social anxiety: the mediation effect of meta-worry. Curr. Psychol. 42, 21489–21498. doi: 10.1007/s12144-022-03229-1

[ref97] Yuan R. M. Peng W. Y. Jiang J. (2024). Relationship between growth mindset and self-control amongst Chinese primary school students: a longitudinal study. Psychol. Res. Behav. Manage. 17, 3101–3109. doi: 10.2147/PRBM.S468490, 39247071 PMC11380487

[ref98] Zhang J. Kuusisto E. Tirri K. (2020). Same mindset, different pedagogical strategies: a case study comparing Chinese and Finnish teachers. Int. J. Learning Teaching Educ. Res. 19, 248–263. doi: 10.26803/ijlter.19.2.15

[ref99] Zhang J. Wang F. King R. B. (2025b). Beyond intelligence: exploring the role of growth mindsets in the domain of social–emotional skills. Br. J. Educ. Psychol. doi: 10.1111/bjep.70037, 41035377 PMC13155037

[ref100] Zhang J. Zhang H. Shang K. Huang Z. (2025a). What matters for students’ social and emotional skills: the associations with student, parent, and teacher growth mindsets? Learning Ind. Diff. 12:102799. doi: 10.1016/j.lindif.2025.102799

[ref101] Zhao S. Du H. Li Q. Wu Q. Chi P. (2021). Growth mindset of socioeconomic status boosts subjective well-being: a longitudinal study. Personal. Individ. Differ. 168:110301. doi: 10.1016/j.paid.2020.110301

[ref102] Zhao Y. F. Zhai X. P. Zhang G. X. Liang X. Xin S. F. (2022). The relationship between growth mindset and perseverance: the mediating role of future time perspective and achievement motivation. Psychol. Dev. Educ. 38, 216–222. doi: 10.16187/j.cnki.issn1001-4918.2022.02.08

[ref103] Zheng T. Y. (2017). A Survey and Study on the current Situation of social Emotional Learning among middle school Students in Southern Taiwan [Master's thesis]. Kaohsiung: National Kaohsiung Normal University.

[ref104] Zhu S. Hu Y. Qi D. Qin N. Chi X. Luo J. . (2023). Single-session intervention on growth mindset on negative emotions for university student mental health (U-SIGMA): a protocol of two-armed randomized controlled trial. Trials 24:713. doi: 10.1186/s13063-023-07748-5, 37940965 PMC10631141

